# YML018C protein localizes to the vacuolar membrane independently of Atg27p

**DOI:** 10.17912/micropub.biology.000391

**Published:** 2021-05-12

**Authors:** Candyce M. Sturgeon, Nicholas Zanghi, Hannah M. Smith, Emily K. Davis, Meaghan R. Robinson, Elizabeth Cabrera, Molly C. Holbrook, Verónica A. Segarra

**Affiliations:** 1 Department of Biology, High Point University, High Point, NC, USA 27268; 2 Department of Biological Sciences, University of North Carolina, Charlotte, USA 28223

## Abstract

The function of the budding yeast YML018C protein remains to be determined. High-throughput studies have reported that the YML018C protein localizes to the vacuolar membrane and physically interacts with the autophagy-related protein Atg27p. While this evidence suggests a potential role for this uncharacterized protein in the process of autophagy, the function of this putative interaction remains uncharacterized. In this micropublication, we report our finding that the localization of the YML018C protein to the vacuolar membrane does not require Atg27p.

**Figure 1. YML018C protein localizes to the vacuolar membrane independently of Atg27p.**
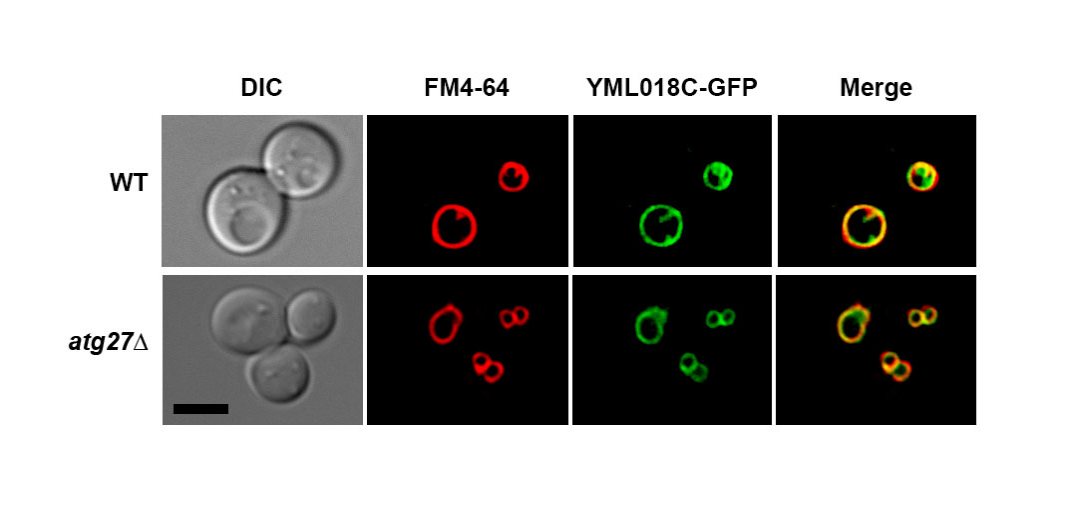
The YML018C protein localizes to the vacuolar membrane in both wild type and *atg27Δ* cells. All cells examined exhibited YML018C at the vacuolar membrane. Scale bar = 5 microns.

## Description

Large-scale studies have localized the uncharacterized YML018C protein to the vacuolar membrane and have identified the autophagy-related protein Atg27p as one of its 10 physical interactors (Huh *et al.*, 2003; Tarasov *et al.*, 2008). These two findings suggested a potential role for the YML018C protein in the process of autophagy. *In silico* methods predict that the YML018C protein has 8 transmembrane domains, with both N- and C-terminal regions facing the cytoplasm (Krogh *et al.*, 2000). Structure prediction algorithms identify the YML018C protein as a candidate GDP-mannose (or nucleotide sugar) transporter (Kelley *et al.*, 2015).

Atg27p is a single-pass transmembrane protein that is important in the catabolic process of autophagy (Segarra *et al.*, 2015). Autophagy is a cellular method of recycling that becomes activated when the cell is subjected to stresses like starvation. Upon the induction of autophagy, a membrane trafficking response ensues that coordinates the formation of large, double-bilayered vesicles called autophagosomes. Autophagosomes are then targeted for docking and fusion to the degradative organelle of the cell, the vacuole in yeast. Nascent autophagosomes sequester unneeded or damaged cellular components for eventual delivery to the vacuole for breakdown and recycling. Atg27p is found in the membranes of cellular compartments that are relevant to autophagy, including the pre-autophagosomal structure (PAS), which serves as the organizing center for autophagosome formation, as well as the Golgi apparatus, early/late endosomes, and the vacuolar membrane (Segarra *et al.*, 2015; Suzuki and Emr, 2018). This complex localization pattern may reflect the role of Atg27p in facilitating the movement of cargoes destined for autophagy from one cellular compartment to another. In fact, it has been shown that deletion of *ATG27* alters the localization of another autophagy-related protein—-Atg9p (Segarra *et al.*, 2015; Yen et. al., 2007). For example, in cells deleted for *ATG27*, the ability of the 6-transmembrane domain protein Atg9p to cycle through the PAS is decreased, even during logarithmic growth, when autophagy is not actively induced by cells. We hypothesized that the YML018C protein might similarly exhibit Atg27p dependency in its localization during logarithmic growth.

Since the YML018C protein and Atg27p both localize to the vacuolar membrane at steady state and reportedly physically interact with one another, we set out to determine whether *ATG27* deletion would trigger a defect in YML018C protein localization. We found that the vacuolar membrane localization of YML018C protein remains intact in atg27*Δ* cells deleted for *ATG27*. This finding indicates that, while the YML018C protein and Atg27p may indeed interact, this physical association is not required for YML018C protein vacuolar membrane localization. This was an initial attempt at elucidating the function of the reported interaction between the YML018C and Atg27p proteins. Further experiments will be needed to determine how these two molecules depend on one another.

## Methods

**Yeast and plasmid methods**

Standard methods were used for genetic manipulations and growth of yeast (Guthrie and Fink 1991). *Saccharomyces cerevisiae* strains used in this study are listed in the table below. Unless otherwise specified, yeast strains were constructed using the Longtine method and their genotypes confirmed using polymerase chain reaction (PCR) and live cell microscopy (Longtine *et al.* 1998).

**Yeast strains used in this study**

**Table:** 

**Name**	**Alias**	**Genotype**	**Reference**
VS54	*YML018C-GFP*	*MATa YML018C-GFP::HISMX6*	Huh *et al.*, 2003
VS85	*YML018C-GFP atg27Δ*	*MATa YML018C-GFP::HISMX6 atg27Δ::HISMX6*	This Paper

**Microscopy methods**

Yeast cells in logarithmic growth phase were mounted in growth medium, and z-stacks were collected at 0.25-µm increments on a DeltaVision elite workstation (Cytiva) based on an inverted microscope (IX-70; Olympus) using a 100×1.4NA oil immersion lens. Images were captured at 24°C with a 12-bit charge-coupled device camera (CoolSnap HQ; Photometrics) and deconvolved using the iterative-constrained algorithm and the measured point spread function. Image analysis and preparation was done using Softworx 6.5 (Cytiva). Vacuolar membrane staining with FM4-64 was carried out as described previously (Segarra *et al.* 2015).
